# The Scheme for Raising Funds at Beckett Hospital, Barnsley

**Published:** 1914-01-03

**Authors:** Alfred Whitham

**Affiliations:** Deputy Chairman House Committee


### The Scheme for Raising Funds at Beckett Hospital, Barnsley.

BY ALFRED WHITHAM, DEPUTY CHAIRMAN HOUSE COMMITTEE.

The principal scheme of decorations here this year is neatness combined with artistic proportion—simple but effective. The day for heavy festoons of evergreens is now past as far as ward decorations are concerned, for experience teaches that such are not only very dangerous, but too "smutty" for nice clean wards. A splendid idea is to have a colour scheme for each separate ward. This is easily arranged by light artificial decorations, assisted with natural flowers placed at convenient stands, with corresponding coloured shades over electric burners between each bed—not over them. Now as to cost. I quite agree with you that nothing should fall on the shoulders of the resident staff or nurses. For years and years no difficulty has been experienced here. I believe the people in the North are far ahead in these matters of our friends in the Southern Counties.

In the first place, a word as to how school children help our hospitals. In many of our schools every child attending the infants' department put something on the Christmas-tree that is provided inside the schools—all sorts of little gifts made by the children themselves. At the "breaking-up" day for Christmas holidays the parents and friends are invited to the school to see what their little boy or girl does in school hours, and the proceedings are usually graced with the presence of the Mayor and Mayoress for the time being, along with members of representative bodies. Some of the children receive prizes that day; all of them give something, and their gifts go to gladden the hearts of the young sufferers in the children's ward of the hospitals in the town and district. The Mayoress and matrons receive these gifts from the children, and to each gift is attached a neat little note from the scholar with these words written on, "Dear Little Friend, I wish you a Merry Christmas." This is an object-lesson, and encourages the children to grow up with a spirit of helping one another in time of need.

The school children are rewarded during the following summer months by being invited in suitable batches to visit the children in the hospital and having afternoon tea with the matron on the tennis lawn. The children are delighted with these arrangements, for after spending a very happy afternoon with their crippled and sick friends they return back to school full of renewed vigour and good wishes to further help the hospital cause next Christmas following.

In addition to the above help we have the usual appeal by means of a short letter in the local papers signed by the matron; one gentleman alone makes himself responsible for the whole of the turkeys, say, about a dozen, for the Christmas dinner; others are responsible for collection boxes. Some are placed in working men's clubs and at collieries. These are very much patronised, and sums varying from ten or twelve shillings up to as much as £7 are brought in from each. In this way we are enabled to give each patient and members of the staff suitable gifts of clothing and all of the children something useful, as well as dolls, etc. The people are very generous to us in our district. On Boxing Day the gifts are distributed by the Mayor and Mayoress, interspersed with music and songs, to which are invited the whole of the friends who have in any way contributed towards the funds.

